# Locally recurrent subcutaneous and muscular hydatid cysts of the leg: an unusual case report

**DOI:** 10.11604/pamj.2015.21.282.6767

**Published:** 2015-08-13

**Authors:** Redouane Ouakrim, Zouhir Amziane, Ouchrif younes, Issam Eloukili, Mohammed Kharmaz, Moulay Omar Lamrani, Ahmed Elbardouni, Mustapha Mahfoud, Berrada Mohammed Saleh

**Affiliations:** 1 Department of Traumatology, Hospital University Avicenna, University Mohammed V, Rabat, Morocco

**Keywords:** Recurrent, subcutaneous hydatid cyst, muscular hydatid cyst, radical excision, anthelmanthic chemotherapy

## Abstract

We report a rare case of 50-year-old Moroccan woman with local recurrence of a subcutaneous hydatid cyst in proximity to the medial surface of the tibia and another cyst at the tibialis posterior muscle in the absence of liver, lung und spleen involvement. The first surgery was done in another hospital three years ago; no adjuvant treatment was performed after surgery. Recurrence was diagnosed according to the MRI appearance, serological and pathological findings. The patient underwent complete excision of the subcutaneous cyst with two centimeters of the medial gastrocnemius muscle; the tibialis posterior muscle cyst was intraoperatively drained and irrigated with scolicidal agent as it was next to the posterior tibial pedicle. A periopertive anthelmintic chemotherapy was administered. Two years after the patient showed no recurrence. This case report and literature review describe an approach to the diagnosis and management of this pathological entity.

## Introduction

Echinococcosis has its highest prevalence in countries, where the common intermediate hosts, sheep and cattle, are raised (such as Middle East, Mediterranean region, Central Europe, Australia and South America) [1, 2]. The incidence of musculoskeletal echinococcosis including involvement of subcutaneous tissue is 1%-5.4% among all cases of hydatid disease [2]. In this report, we present a case of recurrent hydatid cysts involving the medial surface of the tibia and the tibialis posterior muscle. The presented case is distinguished by the simultaneous involvement of subcutaneous tissue and muscle, which remains a rare association.

## Patient and observation

**Figure 1:**
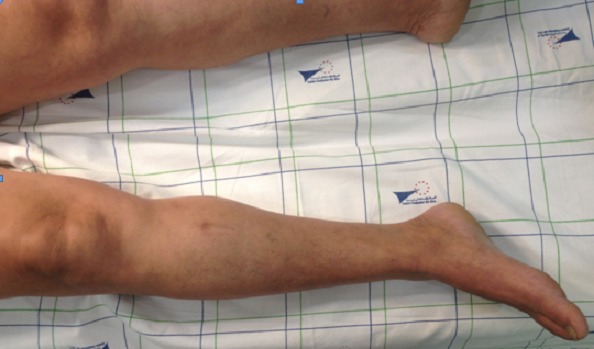
The arrow indicates the swollen area of the right leg

**Figure 2:**
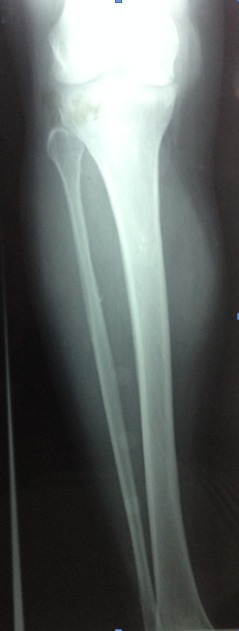
X-ray of the right leg: increased density of soft tissue in the medial aspect of the tibia

**Figure 3:**
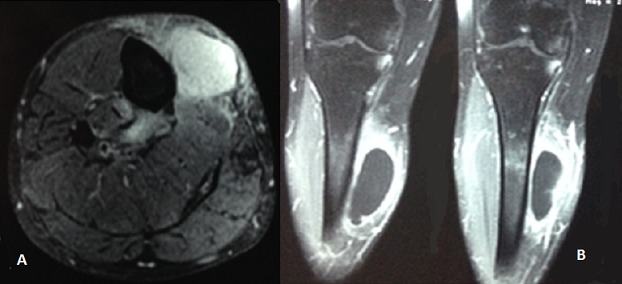
MRI of the right leg, (A) axial T2-weighted: subcutaneous and tibialis posterior cysts; (B) coronal T1 FAT SAT GADO: subcutaneous cystic structure.

**Figure 4:**
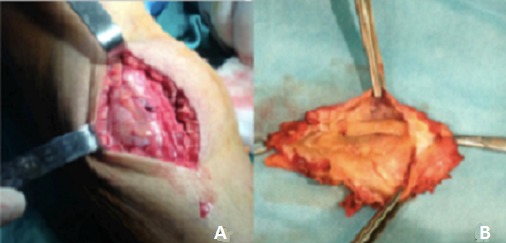
(A) the red arrow marks the subcutaneous hydatid cyst; (B) view of the resected cyst

A 50 year old Moroccan female patient of rural origin presented in Avicenna hospital Rabat-Morocco with history of pain and swelling of the medial aspect of the right leg for two years. The patient had been operated, in another hospital, three years earlier for echinococcosis of the subcutaneous tissue of the right leg with no adjuvant treatment. No history of trauma, fever and weight loss was reported. A painful mass (Figure 1) next the previous incision was palpated on the medial side of the right leg; it was 5×7cm in diameter and there was no erythema and no sign of neurovascular compression, recurrence of hydatid disease was suspected. X-ray of the right leg showed an increased density of soft tissue in the medial aspect of the tibia (Figure 2). MRI (Magnetic Resonance Imaging) revealed the presence of a subcutaneous collection in proximity to the medial surface of the tibia at the junction of his upper and middle third adherent to the periosteum with a thick and scalloped wall, enhanced after gadolinium injection and fluid filled measuring 52×28×72 mm and a second deep collection at the tibialis posterior muscle measuring 21×9×38 mm sitting in front of the posterior tibial and peroneal arteries (Figure 3). Chest X-ray and abdominal ultrasonography did not reveal any abnormality. Complete blood count and electrolytes was normal, ELISA (Enzyme-linked immunosorbent assay) test for echinococcosis was positive. The diagnosis of recurrent hydatidosis has been retained. A three stages Therapeutic protocol was carried out in: Preoperative Albendazole chemotherapy for one month was performed followed by a complete surgical excision of subcutaneous cyst with two-centimeter margin of medial gastrocnemius and intraoperative drainage of the tibialis posterior muscle cyst with hydrogen peroxide irrigation of its pouch as it was adherent to the posterior tibial artery (Figure 4 A;B). Pathological examination of the cyst confirmed the diagnosis. Adjuvant Albendazole chemotherapy was prescribed postoperatively for one year. At two years follow up, no sign of local or distant recurrence was noted and the patient sill under medical surveillance each six months.

## Discussion

Recurrent disease is defined as the appearance of new active cysts after therapy. It occurs after surgical or radiologic intervention and manifests as reappearance of live cysts at the site of a previously treated cyst or the appearance of new disease resulting from procedure-related spillage [3]. The reason of recurrence may reflect the failure to remove all viable cysts and protoscolises at the time of the first operation. Some predictive factors for hepatic cysts was reported to optimise surgical management and to implement preventive measures as rural origin of patients, the voluminous cysts larger than 7cm and unilocular hydatid cyst [4], but no similar study has been performed for musculoskeletal localization. Recommended methods for recurrence diagnosis in addition to clinical and serologic evaluation are ultrasonography, CT (scanning Computed Tomography) or MRI with follow up for at least 3 years [3, 5]. The rarity of the disease does no permit to compare the effectiveness of the various treatment options regarding recurrence rates. Radical surgery (total pericystectomy) with perioperative treatment with benzimedazoles remain the most recommended treatment protocol, however simple drainage with partial cystectomy may be a reasonable therapeutic option when the cyst wall involves major pedicles as in our case [3, 5]. It was recommended to try a treatment with Albendazole alone with recurrent disease if it has not been used previously, in patients refusing surgery, patient at the extremes of age and pregnant women [6, 7]. Other options include percutaneous treatment under ultrasound guidance with needle aspiration and irrigation (PAIR: puncture, aspiration, injection, reaspiration) of scolicidal solutions, as well as medical treatment with the use of Albendazole [8]. Our therapeutic protocol in this case was justified by the pain experienced by the patient and the risk of neuro-vascular compression as it was reported in some case [9].

## Conclusion

Determining the ideal therapeutic approach for a recurrent subcutaneous and muscular hydatid cyst can be quite challenging for the surgeon. Moreover, the rarity of the disease renders the decision making on the favorable treatment quite difficult. Nevertheless, once the diagnosis is established, the surgeon should consider performing a radical procedure with periopertive administration of anthelmathic chemotherapy aiming in minimizing the possibility of a recurrence. A long term follow-up is required in all cases.
